# Atopic Comorbidities and Topical Steroids in Early Childhood Atopic Dermatitis: Are We Missing a Piece of the Puzzle?

**DOI:** 10.1007/s12016-025-09131-5

**Published:** 2026-01-27

**Authors:** Courtney A. Chau, Sonya L. Cyr, Ruchi Gupta, Peter Lio

**Affiliations:** 1 Icahn School of Medicine at Mount Sinai, New York, NY USA; 2 Regeneron Pharmaceuticals Inc, Sleepy Hollow, NY USA; 3 Center for Food Allergy & Asthma Research, Institute for Public Health and Medicine, Northwestern University Feinberg School of Medicine, Chicago, IL USA; 4 Ann & Robert H. Lurie Children’s Hospital of Chicago, Chicago, IL USA; 5 Northwestern University Feinberg School of Medicine, 363 W. Erie Street, Suite #350, Chicago, IL 60654 USA

**Keywords:** Atopic dermatitis, Topical steroids, Food allergies, Type 2 inflammation, Th2

## Abstract

Topical corticosteroids (TCS) remain the first-line treatment for (AD). This narrative review examines clinical data on the immunomodulatory effects of TCS and recent treatments for atopic dermatitis (AD) in early childhood in the context of atopic comorbidities. While TCS are effective in reducing several markers of inflammation in infants with AD, certain type 2 cytokines, such as interleukin (IL)−4, IL-5, and IL-13, remain uncontrolled in the infant stratum corneum, a major source of dysregulated systemic cytokines. A pathomechanism known as remote priming has been identified, wherein allergen-induced antigen-specific immune responses in disrupted skin facilitate allergen-sensitizing immune responses in distant mucosal barrier sites, such as the gut or lung. In the gut, remote priming occurs through the activation of epidermal type 2 innate lymphoid cells (ILC2), which prime gut mast cells and promote food allergies. ILC2s produce IL-5 and IL-13, which remain present in the epidermis with TCS. Consequently, ILC2 cells in the skin of children with AD may remain able to contribute to remote gut priming. Type 2 cytokines also promote immunoglobulin E (IgE) production—a marker of atopic development in children. Over 16 weeks, children aged 6 months to 5 years treated with dupilumab and TCS show a 70% reduction in IgE levels, whereas those treated with TCS alone exhibit a 30% increase in IgE levels. These observations highlight the need for a more comprehensive approach to managing infant AD. Therapies that target inflammation originating from both innate and adaptive effector cells involved in type 2 inflammation may not only alleviate the burden of AD but also disrupt the progression of the atopic march.

## Introduction

Since the introduction of topical corticosteroids (TCS) in the 1950 s, TCS have remained the first-line therapy for atopic dermatitis (AD), providing effective relief from its signs and symptoms [1, 2]. Recent evidence suggests that TCS not only alleviate symptoms but also help normalize various skin and systemic inflammatory markers in infant AD, supporting their role in short-term disease control [3]. Pediatric atopic dermatitis is closely associated with an increased risk of other atopic diseases, including allergic rhinitis, asthma, and food allergies [4–6]. These conditions share an underlying type 2 inflammatory pathway, which often initially manifests in early childhood in the skin as AD. This connection underscores the importance of a holistic approach to managing AD, as it may play a critical role in preventing the progression to other atopic disorders—a phenomenon traditionally referred to as the “atopic march” [7].

Advances in understanding the systemic and epidermal innate immune pathways, as well as memory pathways that contribute to the dysregulated type 2 inflammatory signals in AD offer new insights. These developments raise the question of whether TCS adequately address the key factors driving AD and other diseases within the atopic diathesis. This non-systematic, narrative review examines clinical evidence, which focuses on the key innate and memory components driving type 2 inflammation in early childhood AD, their role in the pathomechanism of remote priming this population, and systemic and epidermal immunomodulation following treatment with TCS. It also reviews pivotal clinical evidence on the relationship between food allergies and the management of infant AD using TCS or barrier enhancement therapy. Finally, it integrates clinical findings on the immunomodulatory effects of existing and emerging treatments to deepen understanding of therapeutic options and their broader impact on managing AD and related atopic conditions.

### Type 2 Inflammation: a Puzzle Built around Two Major Parts: ILC2 and Th2 Cells

Type 2 inflammation encompasses both innate and memory immune responses that interact in self-reinforcing loops driven predominantly by key type 2 cytokines. These cytokines are primarily produced by innate lymphoid type 2 cells (ILC2s) and memory type 2 helper T cells (Th2) [8]. At epithelial surfaces, alarmins—including thymic stromal lymphopoietin (TSLP), interleukin (IL)−25, and IL-33—activate tissue-resident immune cells such as dendritic cells, ILC2s, and mast cells [9]. This activation initiates a cascade that recruits circulating granulocytes—particularly eosinophils and basophils—and stimulates sensory neurons, leading to itch. ILC2s are characterized by robust production of IL-5 and IL-13, which contribute to eosinophil recruitment, sensory neuron activation, and T-helper cells polarization. Th2 cells, which produce IL-4, IL-5, IL-9, IL-13, and IL-31 in response to antigen-specific activation, further amplify granulocyte recruitment and perpetuate type 2 inflammation [10].

IL-4, IL-5 and IL-13 are dominant signaling molecules, common to both ILC2 and Th2 cells. IL-4 and IL-13 synergistically play critical roles in inducing isotype switching to IgE production in B cells and regulating the proliferation of IgE-producing B cells [10]. IgE production leads to allergen sensitization and plays a pivotal role in atopic comorbidity development [11].

### From Skin to Gut: The Role of Type 2 Inflammatory Cells in Remote Priming and Food Allergy Development in Infants with AD

At birth, innate immunity carries most of the burden because adaptive memory is minimal and maternal antibodies wane over the first months of life. Innate lymphoid cells are therefore relatively abundant and particularly active in early life, especially at barrier tissues. Neonatal leukocytes naturally tend towards regulatory or Th2-skewing cytokine to support survival during the transition from the intrauterine environment to the microbe-rich world. Barrier tissues, along with epithelial-derived alarmins and ILC2s, mast cells, eosinophils, monocytes, and dendritic cells, together determine whether antigen encounters occur in a context of danger or a steady-state, tolerogenic environment. This process informs type 2 allergic pathway activation. Thus, ILCs play a pivotal role at the intersection of innate and adaptive immunity, helping to “educate” the developing immune system on how to interpret microbial and environmental signals [12].

**Fig. 1:**
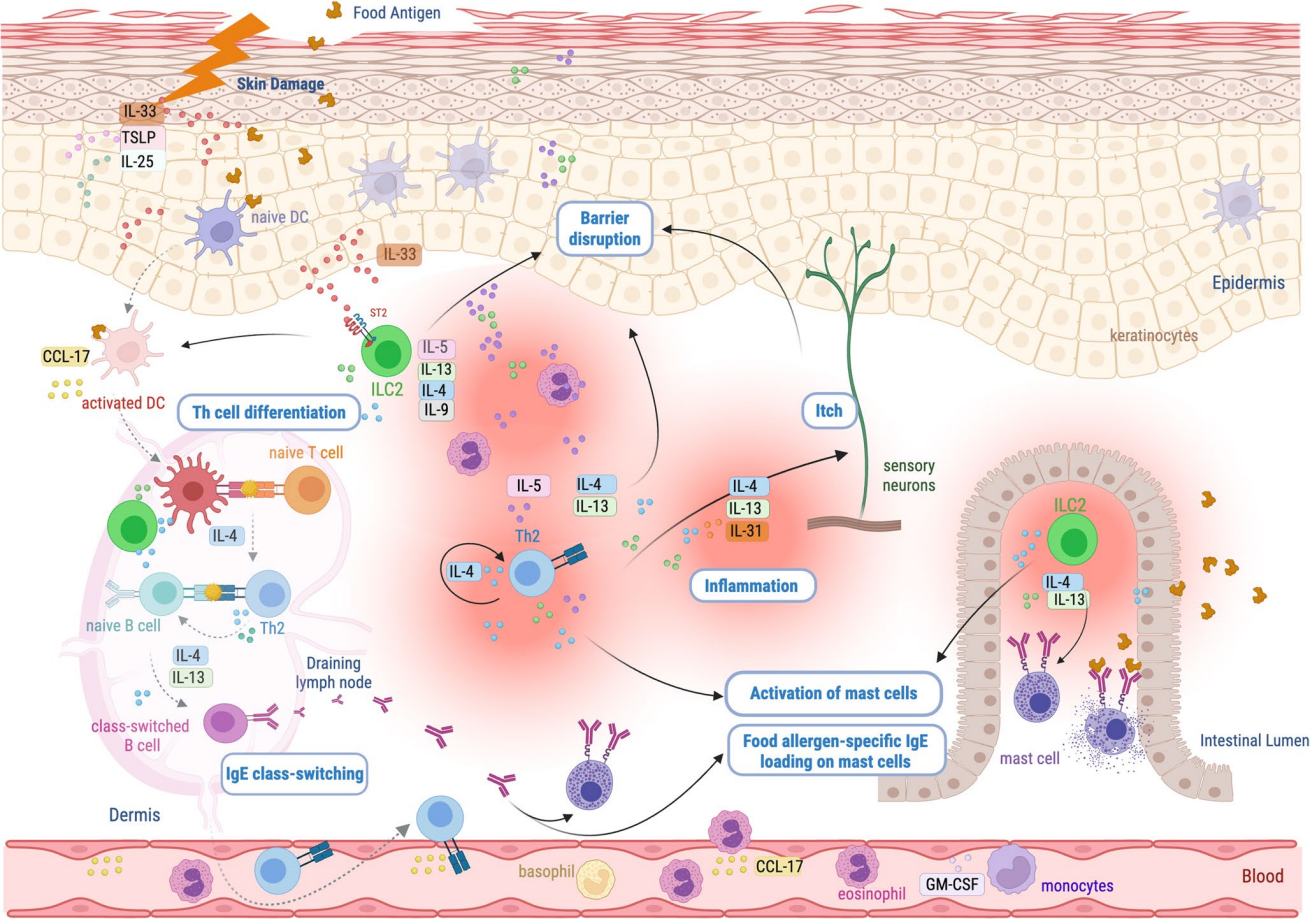
Graphical depiction of local and remote priming. Infant epicutaneous sensitization, or local priming, can occur through a combination epidermal damage, type 2 signaling and food antigen exposure. Epidermal damage is associated with alarmin signals (IL-33, IL-25 and TLSP) and ILC2 cytokines. Together, these promote DCs to educate memory T and B lymphocytes in a ‘danger’ context, thereby recognizing food antigens as allergens, resulting in the loading of skin-resident mast cells with allergen-specific antibodies. DCs and tissues also produce chemotactic agents such as CCL-17 enhancing the recruitment of type 2 effector cells from the circulation. Remote gut priming is thought to be initiated primarily with epidermal IL-33 binding of ST2 on ILC2s in a nonredundant manner for optimal distal IgE production. The role of ILC2s in this process may involve distinct mechanisms; 1-upstream of effector T cell generation by influencing DC migration or activation, 2-contribution to mast cell expansion in the intestine and 3-within lymphoid organs to complement the IL-4 and IL-13 primarily derived from T follicular helper (TFH) cells, thereby further enhancing B cell class switching to IgE. Gut resident mast cells are ultimately loaded with food-allergen specific IgE, primed for food allergen recognition and degranulation upon subsequent oral exposure. Created in Biorender [21]

In AD, barrier damage triggers keratinocyte production of type 2-promoting mediators, including IL-25, TSLP, and IL-33, which activate basophils, innate lymphoid cells, and dendritic cells, perpetuating skin barrier dysfunction and amplifying Th2 immune responses [13]. In the setting of a defective skin barrier, antigen exposure within an epidermal type 2 inflammatory milieu promotes IgE production against specific antigens, a process called epicutaneous sensitization, or local priming [14, 15]. Exposure to food antigens through a damaged skin barrier can drive FA via epicutaneous sensitization, whereas oral exposure promotes tolerance and protection against FA development [14, 16–18]. The concept of remote priming has been proposed to explain the link between AD and FA [19, 20]. In this model, allergen exposure through a disrupted skin barrier induces antigen-specific immune responses that promote sensitization at distant mucosal sites, including the gut and lungs. Epidermal damage-driven IL-33 activates ILC2s which facilitate remote priming (Fig. 1) by enhancing dendritic cell migration and T cell priming, as well as by promoting IL-4 and IL-13-dependent mast cell expansion and IgE class switiching within gut associated lymphoid tissues.

### Systemic and Epidermal Immunomodulation Induced by TCS Therapy in Infant AD

McAleer et al. (2021) found that TCS treatment in infants with AD resulted in significant changes in systemic and skin biomarkers, with the implication that the skin barrier contributes to immune dysregulation in infant AD. Their study assessed 74 infants (mean age of 7 months) with AD following 6 weeks of topical corticosteroid therapy, focusing on peripheral blood and skin-derived (stratum corneum, SC) biomarkers. Plasma samples from 47 patients and 20 controls were analyzed for immune biomarkers. Additionally, SC samples were examined for natural moisturizing factors (NMF) in 74 patients and 18 controls, while immune biomarkers were evaluated in 66 patient samples and 13 controls [3].

In the plasma, biomarkers [C-C motif chemokine(CCL)17, IL-13, CCL22, IL-5 and CCL26] and vascular cell adhesion markers (soluble intercellular adhesion molecule 1 and soluble vascular cell adhesion molecule 1) decreased after therapy, suggesting that TCS contributes to reducing these systemic markers related to type 2 inflammation after 6 weeks of treatment. The most prominent change in the SC was a decrease in transepithelial water loss (TEWL), and a decrease in innate inflammation markers IL-18 and IL-8, as well as angiogenesis and vascular markers (Flt-1 and vascular endothelial growth factor-A). In contrast, IL-5 was increased, with no change in IL-13, which remained elevated [3]. Although the study did not investigate the cellular sources of these cytokines, this observation could suggest that ILC2 remains active locally within the skin during TCS use, despite a decrease in circulating type 2 inflammation markers and some innate inflammation markers. Alternatively, other cytokines and chemokines increased in the epidermis following TCS treatment in this population, including GM-CSF, CCL13, CCL4, CCL3, IL-15, and IL-1α, which are associated with immune cell recruitment, inflammation, and tissue repair. As such, these could contribute to maintaining activated epidermal type 2 inflammation, including eosinophils as a potential source of IL-5. Of note, the trial showed no improvement in natural moisturizing factor (NMF) after TCS therapy, irrespective of the presence of filaggrin gene mutations. Therefore, while topical corticosteroids are effective in treating AD, the epidermal increase in IL-5 and unaffected IL-13, combined with other increased inflammatory markers, suggest the persistent presence of epidermal type 2 inflammation, including active ILC2s, which have been associated with epicutaneous sensitization leading to FA.

### Food Allergies and Infant AD: Clinical Evidence Following TCS and Barrier Enhancement Therapies

Clinical evidence suggests that corticosteroid use in infants with AD does not reliably prevent the development of FA. However, interpretation is limited by confounding from disease severity, as direct comparisons of FA outcomes between severity-matched patients treated with TCS and untreated patients are lacking. In a cohort of 4453 infants, Martin et al. (2014) reported that infants with eczema were 11-fold more likely to develop peanut allergy and 5.8-fold more likely to develop egg allergy, reinforcing the strong association between these two conditions. They further demonstrated FA prevalence increased with AD severity, paralleling the need for topical corticosteroid treatment. The highest risk of FA was observed in infants with eczema diagnosed within the first three months of life who required prescription topical corticosteroids, among whom food allergy prevalence reached 50.8% [22]. Consistent with this, a recent systematic review and meta-analysis reported FA prevalence of approximately 33% in mild AD and 52% in severe AD [23]. Together, these findings support a strong association between severe AD and FA, likely reflecting greater underlying barrier dysfunction, epidermal inflammation, and immune dysregulation. Although TCS can restore visible barrier integrity in the short term, they may not fully address underlying innate inflammatory pathways that contribute to FA risk.

The impact of proactive vs. reactive TCS use in infants 7–13 weeks old with mild AD who develop FA has been evaluated (median EASI score 4, highest score of 8 on a scale of 0–72) [24]. Proactive TCS treatment, defined as application to lesional and nonlesional skin, or reactive TCS treatment, defined as application to lesional skin only, were compared to determine FA incidence at 28 weeks. Proactive management with TCS resulted in a reduced proportion of patients with FA (31.4%) compared to reactive therapy TCS (41.0%). The authors propose that proactive TCS application on a larger area including nonlesional skin where subclinical inflammation exists may also work on suppressing IgE sensitization and FA. While this may be the case, the proportion of patients with hen’s egg white IgE sensitization increased in both groups: at baseline vs. end of treatment in the proactive group: 9.5% vs. 44.9%, and in the reactive group: 8.4% vs. 52.5%. Moreover, compared with expected rates of FA in mild AD, proactive TCS management did not reduce food allergy incidence (31.4% vs. 33% reported in meta-analysis [23]). In contrast, infants managed with reactive TCS exhibited a higher-than-expected FA prevalence (41% vs. 33%). While this raises the possibility that reactive TCS use may contribute to increased FA risk, definitive conclusions are limited by the absence of severity-matched comparisons between TCS-treated and untreated patients. Notably, the authors do not recommend proactive TCS therapy, as infants in this group demonstrated reduced gains in body weight and height by the age of 28 weeks of age compared with those on reactive therapy.

The therapeutic benefit of applying a physical protective layer to the disrupted barrier is thoroughly investigated. The barrier enhancement for eczema prevention (BEEP) trial evaluated whether daily use of emollient in the first year could prevent AD in high-risk children [25]. Newborns were randomized to a moisturizer or control group and followed for the development of AD by 2 years of age. Eczema developed at similar rates in both groups, suggesting that inflammation, rather than barrier disruption alone, may be the primary driver of pathophysiology. In contrast to emollients, TCS address aspects of type 2 inflammation and may indirectly support some elements of barrier repair. Alternatively, the authors proposed that topical application could exacerbate epicutaneous sensitization ‘trapping’ allergens within the skin, a mechanism that could also occur with TCS use.

A clinical trial of topical pimecrolimus, a calcineurin inhibitor, has provided valuable insights into the management of AD and its associated comorbidities. The Study of the Atopic March aimed to assess whether early intervention with pimecrolimus could limit the progression of the atopic march in infants with AD, while also evaluating its efficacy and safety [26]. Infants ages 3 to 18 months with recent-onset AD (≤ 3 months) were observed for a mean of 2.8 years (*N* = 1,091). Pimecrolimus functions by preventing the activation of T-cells and the release of inflammatory cytokines, thereby reducing inflammation. The trial demonstrated that while pimecrolimus effectively managed AD symptoms, it had no impact on the development of atopic comorbidities. Although data is lacking in pediatric patients, a study in adults demonstrates that pimecrolimus has minimal effects on IgE levels [27]. The study highlighted that infants with more severe AD were at greater risk of developing atopic comorbidities. Given that the calcineurin pathway is not known to play a direct role in ILC2 activation or function, these results could support the hypothesis that ILC2s may play a critical role in linking AD severity to the progression of atopic conditions [28].

### Existing and Emerging Therapies for Early Childhood AD: Clinical Biomarkers and Immunomodulatory Effects of Nonsteroidal Treatments

Treatment-induced immunomodulation data in young children are available for dupilumab, which is approved for treating AD in infants aged 6 months and older. The biologics tralokinumab and lebrikizumab, currently in clinical trials for pediatric patients aged 6 months to 12 years, have immunomodulation data that is primarily available from studies conducted in adults, as pediatric-specific data is still being generated. The approved topical PDE-4 inhibitors and Aryl hydrocarbon receptor (AhR) agonist for young children have available immunomodulation data from older age groups treated with these agents. Finally, a topical JAK inhibitor, ruxolitinib, is approved for age 2 years and above, while oral formulations of upadacitinib, abrocitinib and baricitinib, outside of the US, are being evaluated in younger children for AD. Most insights into their effects on type 2 inflammation immunomodulation come from adult studies.

#### Dupilumab

Dupilumab is a monoclonal antibody targeting the IL-4Ra, thereby inhibiting IL-4 and IL-13 signaling. Treatment with dupilumab has been associated with clinical improvement of moderate to severe AD in several clinical trials including infants as young as 6 months old [29–33]. Meta-analyses using real world evidence suggest that children treated with dupilumab are at reduced risk of atopic comorbidities [34, 35]. These observations support the hypothesis that enhanced IL-4 and IL-13 cytokine signaling coordinates innate and adaptive type 2 immune responses, promotes IgE production, and thus may drive atopic progression.

In a 16-week trial of dupilumab in children aged 6 months to 6 years, participants received TCS with dupilumab or placebo. While the mean serum IgE levels were reduced by 70% in those treated with dupilumab and TCS, they were significantly *increased* by 30% in participants treated with placebo and TCS. Although patients on placebo and TCS saw moderate improvements in clinical disease severity as measured by EASI, IgE paradoxically increased during the treatment period [36]. As such, functionally, young children managed with TCS alone demonstrate increased IgE synthesis, despite modest clinical improvements, suggesting that TCS do not holistically address the mechanisms underlying type 2 inflammation, and, as such, do not prevent the production of IgE.

Although without a comparison to TCS, a study of 36 pediatric AD patients, 4–17 years, treated with dupilumab showed both a 86.7% (95% CI: 70.2–94.1) decrease in total IgE and a substantial decrease in food allergen specific IgE for 10 common food allergens (peanut, hazelnut, cashew nut, pistachio, almond, walnut, hen’s egg, cow’s milk, kiwi, and apple), ranging from 70.5% to 82.5%, by 1 year of treatment [37]. Similarly, a retrospective study of pediatric AD patients (*n* = 60, 6 months-18 years) with food allergy and treated with dupilumab found decreases of 0.6% (95% CI: −0.8% to −0.4%) in food allergen specific IgE for each month of treatment [38]. As more children are started on dupilumab at an early age, the interaction of dupilumab with the clinical course of FA warrants further investigation.

Another trial randomized pediatric AD patients (2–18 years, *n* = 36) into 3 groups: dupilumab and topical treatment (moisturizers and TCS), cyclosporine and topical treatment, and topical treatment alone. The trial found that IgG^+^CD23hiIL-4Rα + type 2 memory B cells and total IgE were decreased only in the dupilumab-treated group [39].

Beyond IgE, studies have also demonstrated decreases in other markers of type 2 inflammation with dupilumab treatment in the young children population (6 months to 5 years), including serum thymus and activation-regulated chemokine (TARC)/CC chemokine ligand 17 (CCL17), lactate dehydrogenase (LDH), and macrophage-derived chemokine/CCL22 [40–42]. One study found that 16 weeks of dupilumab treatment in 20 pediatric AD patients (aged 3–17 years) resulted in a significant decrease in expression of PARC and TARC in serum and both lesional and non-lesional skin tape strips. In addition, significant reductions in CCL27, IL-8, IL-18, periostin, and MMP-1 levels were observed in skin strips of lesional sites [43]. Overall, clinical evidence in infants and children suggests that dupilumab elicits significant reductions in serum and epidermal inflammation markers, as well as IgE levels.

#### Tralokinumab

Tralokinumab, a monoclonal antibody binding to IL-13, is approved for the treatment of moderate to severe AD in patients 12 years and older [44]. Among tralokinumab-treated adult AD patients in the Phase 3 ECZTRA 1 trial, significant serum reductions in TARC/CCL17 IgE, TARC/CCL17 and periostin were observed at weeks 4, 8, and 16, compared to placebo. A significant reduction in IL-22 was observed between the two groups at week 16 [45].

#### Lebrikizumab

Lebrikizumab is a monoclonal antibody that binds IL-13 and is also approved for treatment in patients above 12 years with moderate-to-severe AD [46]. Among adults with AD treated with lebrikizumab in the ADvocate1 and ADvocate2 trials, reductions in serum periostin and CCL13 were observed at timepoints of 4 weeks and 16 weeks and were significant compared to placebo. Reductions in TARC/CCL17 were observed at 4 weeks, but by 16 weeks, did not differ from placebo. Reductions in IgE were not significant compared to placebo at weeks 4 and 16, but achieved statistical significance with approximately 20% reduction versus baseline levels at week 52 [47].

#### PDE-4 Inhibitors

Crisaborole ointment, 2%, is a topical phosphodiesterase 4 (PDE-4) inhibitor approved for the treatment of mild to moderate AD in infants as young as 3 months of age [48]. Roflumilast is formulated as a cream at differing concentrations and is approved down to 2 years of age, with ongoing trials down to 3 months of age. Inhibition of PDE-4 has demonstrated broad anti-inflammatory effects. It has been shown to contribute to decreases in systemic Th1-, Th2-, and Th17-related cytokines, including IL-5, IL-10, IL-17, and IL-22. Their impacts on serum IgE in AD patients is currently unclear. In cultured B cells from AD patients, PDE activity has been associated with IgE synthesis by mononuclear leukocytes, which is successfully reduced by PDE inhibition [49].

#### Aryl Hydrocarbon Receptor (AhR) Agonists

Tapinarof for atopic dermatitis (AD) is approved for adults and children 2 years of age and older, with ongoing trials down to 3 months of age. While extensively studied for efficacy, changes to systemic or epidermal biomarkers with topical AhR agonist treatment have scarcely been reported for AD patients. AhR signaling in B cells may bias B-cell differentiation toward memory phenotypes and away from antibody-secreting plasma cells, which may impact IgE production [50]. However, clinical data assessing type 2 inflammatory markers or IgE in AD in this context are currently not available.

#### Janus Kinase Inhibitors

Topical ruxolitinib (RUX) 1.5%, a JAK1/JAK2 inhibitor, is approved in non-immunocompromised children as young as 2 years of age for the treatment of mild to moderate AD [51]. In sera collected from adult patients at week 8, TARC/CCL17 levels were reduced (*P* < 0.01) in patients treated with 1.5% RUX cream BID versus vehicle. Total serum IgE levels were numerically reduced in patients treated with 1.5% RUX cream (QD or BID), but the reduction did not reach statistical significance [52].

Upadacitinib works by blocking the Janus kinase 1 (JAK1) signaling molecule, and is approved for the treatment of moderate to severe AD in patients 12 years and older [53]. In one study in adults treated with upadacitinib 15 mg (*n* = 213), IgE significantly increased at week 4 and 48 compared to baseline [54]. In the upadacitinib 30 mg group (*n* = 70), IgE values significantly increased at weeks 24, 36, and 48 compared to baseline. TARC values were not significantly different from baseline.

Baricitinib is an inhibitor of JAK1 and JAK2 signaling molecules, and is approved for moderate to severe AD in patients 2 years and older outside of the US [55]. We did not find type 2 inflammation markers or IgE modulation data for baricitinib.

In adults, oral abrocitinib treatment has been associated with the downregulation of genes associated with inflammation, epidermal hyperplasia, and Th2 and Th22 immune responses in the skin by 12 weeks, compared to placebo [56]. In vitro evaluation of PBMCs showed that abrocitinib induced inhibition of IL-5, IL-13, IL-10, IL-9 and TNFa in the presence of peanut stimulation [57]. A study was recently completed to assess the role for abrocitinib as a potential treatment for food allergy in adult patients, however, the results have not yet been released [58].

#### Implications for Infant AD

AD is commonly diagnosed in early childhood, with approximately 60% of cases identified by one year of age and 90% by five years, often within the first 6 months of life [7, 59]. During this critical developmental window, innate lymphocytes and epithelial immunity play an outsized role compared to later in life, whilst adaptive T-cell immunity is still maturing, and the infant T-cell compartment remains distinct from that of adults for at least the first 2 years of life [60]. Neonatal antigen exposure biases immunity toward Th2 responses, with continued shaping by environmental antigens during infancy [61].

AD in early childhood is a multifactorial arising from the interaction of environmental exposures and type 2 immune responses involving ILC2s, Th2 cells and B cells, with important implications for disease pathogenesis and associated atopic comorbidities, including FA. This review examined the immunomodulatory effects of TCS in infants and young children, which have long been a cornerstone in managing AD. While TCS effectively reduce inflammation and improve visible barrier function, their impact on some key immune pathways, particularly innate drivers of type 2 inflmmation in early life, remains incompletely defined.

The available evidence suggests that TCS may only partially address the innate drivers of type 2 inflammation, limiting their ability to mitigate the risk of FA and other atopic comorbidities. In contrast, emerging therapies, including biologics such as dupilumab, offer a broader immunologic approach by inhibiting shared signaling pathways across innate and adaptive type 2 immunity. Comparative studies indicate that dupilumab not only improves barrier integrity but also modulates biomarkers associated with FA and other atopic conditions in young children, suggesting a benefits beyond cutaneous disease control.

These observations highlight the importance of considering the long-term immunologic consequences of early therapeutic intervention in AD and its potential to alter the trajectory of atopic comorbidities. In young children with AD, the evidence discussed here suggests that controlling both critical arms of type 2 inflammation driven by ILC2 and Th2-cells can reduce IgE sensitization and AD-related atopic comorbidities. Further evaluations are required to determine whether very early intervention could prevent both IgE sensitization as well as AD-related morbidity. Indeed, timely action may meaningfully reduce the burden of FA.

Mechanistic insights from Asrat et al. (2020) further support this concept by identifying long-lived plasma cells (LLPCs) as a durable reservoir for IgE memory generated through repeated allergen exposure in an IL-4Ra-dependent manner [62]. This finding suggests that early interruption of IL-4Rα signaling during allergen sensitization may prevent the establishment of persistent IgE-mediated immunity and subsequent FA.

Once established, LLPCs migrate to bone marrow, where they become IL-4Rα–independent, lose conventional B-cell markers (including BCR and surface IgE), and serve as sustained sources of pathogenic IgE. At this stage, they are largely resistant to existing therapies. Novel approaches, such as BCMA×CD3–based strategies, may offer a means to eliminate LLPCs and reduce established allergen-specific IgE, thereby alleviating the burden of persistent FA [63]. Although the application of such therapies to pediatric FA warrants careful study, emerging data suggest that immune-modulating bispecifics designed for inflammatory diseases may exhibit safety profiles distinct from those developed for oncology [64–66].

## Conclusion

Managing early childhood AD requires a nuanced approach that integrates an understanding of type 2 inflammation, the role of ILC2s, Th2 and B cells, and the interplay between barrier dysfunction, environmental exposure, and FA. Although TCS remain a valuable tool, the findings discussed here suggest that they only partially address type 2 inflammation. In contrast, biologics, such as dupilumab, demonstrate the potential for more targeted and comprehensive treatment strategies. These approaches encompass both innate and adaptive immune components, while also providing downstream regulation of IgE-mediated allergen sensitization. Future research should focus on refining these approaches and addressing the broader implications of early intervention for FA and other atopic comorbidities in young children. Longitudinal pediatric trials directly comparing TCS to early biologic intervention can enhance our understanding of their respective long-term immunomodulatory effects and subsequent comorbidity development. By doing so, we can move closer to a holistic framework for managing AD and its associated conditions in early childhood.

## Data Availability

No datasets were generated or analysed during the current study.

## References

[1] Das A, Panda S (2017) Use of topical corticosteroids in dermatology: an evidence-based approach. Indian J Dermatol 62(3):237–250. 10.4103/ijd.IJD_169_17 28584365 10.4103/ijd.IJD_169_17 PMC5448257

[2] Ch’en PY, Lio PA (2024) Nonsteroidal approaches for Atopic Dermatitis®: a clinical update. Dermatitis® 35(6):596–604. 10.1089/derm.2023.0373 38320243 10.1089/derm.2023.0373

[3] McAleer MA, Jakasa I, Stefanovic N, McLean WHI, Kezic S, Irvine AD (2021) Topical corticosteroids normalize both skin and systemic inflammatory markers in infant atopic dermatitis. Br J Dermatol 185(1):153–163. 10.1111/bjd.19703 33269467 10.1111/bjd.19703 PMC8359435

[4] Mehta Y, Fulmali DG Relationship between atopic dermatitis and food allergy in children. Cureus. 14(12):e33160. 10.7759/cureus.33160 10.7759/cureus.33160 PMC9886409 36726939

[5] Bantz SK, Zhu Z, Zheng T (2014) The Atopic march: progression from Atopic Dermatitis to Allergic Rhinitis and Asthma. J Clin Cell Immunol 5(2):202. 10.4172/2155-9899.1000202 25419479 10.4172/2155-9899.1000202 PMC4240310

[6] Sitarik AR, Eapen AA, Biagini JM et al (2025) Phenotypes of Atopic Dermatitis and development of Allergic Diseases. JAMA Netw Open 8(6):e2515094. 10.1001/jamanetworkopen.2025.15094 40504529 10.1001/jamanetworkopen.2025.15094 PMC12163678

[7] Han H, Roan F, Ziegler SF (2017) The atopic march: current insights into skin barrier dysfunction and epithelial cell-derived cytokines. Immunol Rev 278(1):116–130. 10.1111/imr.12546 28658558 10.1111/imr.12546 PMC5492959

[8] Gurram RK, Zhu J (2019) Orchestration between ILC2s and Th2 cells in shaping type 2 immune responses. Cell Mol Immunol 16(3):225–235. 10.1038/s41423-019-0210-8 30792500 10.1038/s41423-019-0210-8 PMC6460501

[9] Hammad H, Lambrecht BN (2015) Barrier epithelial cells and the control of type 2 immunity. Immunity 43(1):29–40. 10.1016/j.immuni.2015.07.007 26200011 10.1016/j.immuni.2015.07.007

[10] Moniaga CS, Tominaga M, Takamori K (2021) The pathology of type 2 inflammation-associated itch in atopic dermatitis. Diagnostics 11(11):2090. 10.3390/diagnostics11112090 34829437 10.3390/diagnostics11112090 PMC8618746

[11] Greene D, Moore Fried J, Wang J (2025) IgE in allergic diseases. Immunol Rev 334(1):e70057. 10.1111/imr.70057 40862531 10.1111/imr.70057

[12] Yu JC, Khodadadi H, Malik A et al (2018) Innate immunity of neonates and infants. Front Immunol 9:1759. 10.3389/fimmu.2018.01759 30105028 10.3389/fimmu.2018.01759 PMC6077196

[13] Yang G, Seok JK, Kang HC, Cho YY, Lee HS, Lee JY (2020) Skin barrier abnormalities and immune dysfunction in atopic dermatitis. Int J Mol Sci 21(8):2867. 10.3390/ijms21082867 32326002 10.3390/ijms21082867 PMC7215310

[14] Strid J, Strobel S (2005) Skin barrier dysfunction and systemic sensitization to allergens through the skin. Curr Drug Targets-Inflamm Allergy 4(5):531–541. 10.2174/156801005774322199 16248822 10.2174/156801005774322199

[15] Elias PM (2010) Therapeutic implications of a barrier-based pathogenesis of atopic dermatitis. Ann Dermatol 22(3):245–254. 10.5021/ad.2010.22.3.245 20711259 10.5021/ad.2010.22.3.245 PMC2917676

[16] Cook-Mills JM, Emmerson LN (2022) Epithelial barrier regulation, antigen sampling, and food allergy. J Allergy Clin Immunol 150(3):493–502. 10.1016/j.jaci.2022.06.018 35945053 10.1016/j.jaci.2022.06.018

[17] Toit GD, Sayre PH, Roberts G et al (2016) Effect of avoidance on peanut allergy after early peanut consumption. N Engl J Med 374(15):1435–1443. 10.1056/NEJMoa1514209 26942922 10.1056/NEJMoa1514209 PMC12333932

[18] Perkin MR, Logan K, Tseng A et al (2016) Randomized trial of introduction of allergenic foods in breast-fed infants. N Engl J Med 374(18):1733–1743. 10.1056/NEJMoa1514210 26943128 10.1056/NEJMoa1514210

[19] Ubags ND (2020) Remote tissue immune priming in allergic disease. Mucosal Immunol 13(5):719–720. 10.1038/s41385-020-0328-0 32719410 10.1038/s41385-020-0328-0 PMC7434592

[20] Waizman DA, Brown-Soler I, Martin AL et al (2025) Skin damage signals mediate allergic sensitization to spatially unlinked antigen. Sci Immunol. 10.1126/sciimmunol.adn0688 40184440 10.1126/sciimmunol.adn0688 PMC12100540

[21] Cyr S (2025) Local and remote priming in atopic dermatitis: created in biorender. https://BioRender.com/hyw5wbb

[22] Martin PE, Eckert JK, Koplin JJ et al (2015) Which infants with eczema are at risk of food allergy? Results from a population-based cohort. Clin Exp Allergy 45(1):255–264. 10.1111/cea.12406 25210971 10.1111/cea.12406

[23] Christensen MO, Barakji YA, Loft N et al (2023) Prevalence of and association between atopic dermatitis and food sensitivity, food allergy and challenge-proven food allergy: a systematic review and meta-analysis. J Eur Acad Dermatol Venereol 37(5):984–1003. 10.1111/jdv.18919 36695076 10.1111/jdv.18919

[24] Yamamoto-Hanada K, Kobayashi T, Mikami M et al (2023) Enhanced early skin treatment for atopic dermatitis in infants reduces food allergy. J Allergy Clin Immunol 152(1):126–135. 10.1016/j.jaci.2023.03.008 36963619 10.1016/j.jaci.2023.03.008

[25] Chalmers JR, Haines RH, Bradshaw LE et al (2020) Daily emollient during infancy for prevention of eczema: the beep randomised controlled trial. Lancet 395(10228):962–972. 10.1016/S0140-6736(19)32984-8 32087126 10.1016/S0140-6736(19)32984-8 PMC7086156

[26] Schneider L, Hanifin J, Boguniewicz M et al (2016) Study of the atopic march: development of atopic comorbidities. Pediatr Dermatol 33(4):388–398. 10.1111/pde.12867 27273433 10.1111/pde.12867 PMC5649252

[27] Kasai H, Kawasaki H, Fukushima-Nomura A et al (2021) Stratification of atopic dermatitis patients by patterns of response to proactive therapy with topical tacrolimus: low serum IgE levels and inadequately controlled disease activity at the start of treatment predict its failure. Ann Med 53(1):2207–2216. 10.1080/07853890.2021.2004319 10.1080/07853890.2021.2004319 PMC8805968 34797182

[28] Qin M, Fang Y, Zheng Q et al (2024) Tissue microenvironment induces tissue specificity of ILC2. Cell Death Discov 10(1):324. 10.1038/s41420-024-02096-y 39013890 10.1038/s41420-024-02096-y PMC11252336

[29] Simpson EL, Bieber T, Guttman-Yassky E et al (2016) Two phase 3 trials of dupilumab versus placebo in atopic dermatitis. N Engl J Med. 10.1056/NEJMoa1610020 27690741 10.1056/NEJMoa1610020

[30] Blauvelt A, de Bruin-Weller M, Simpson EL, Chen Z, Zhang A, Shumel B (2022) Dupilumab with topical corticosteroids provides rapid and sustained improvement in adults with moderate-to-severe atopic dermatitis across anatomic regions over 52 weeks. Dermatol Ther (Heidelb) 12(1):223–231. 10.1007/s13555-021-00638-1 34806137 10.1007/s13555-021-00638-1 PMC8776906

[31] Simpson EL, Paller AS, Siegfried EC et al (2020) Efficacy and safety of dupilumab in adolescents with uncontrolled moderate to severe atopic dermatitis: A phase 3 randomized clinical trial. JAMA Dermatol 156(1):44–56. 10.1001/jamadermatol.2019.3336 31693077 10.1001/jamadermatol.2019.3336 PMC6865265

[32] Paller AS, Siegfried EC, Thaçi D et al (2020) Efficacy and safety of dupilumab with concomitant topical corticosteroids in children 6 to 11 years old with severe atopic dermatitis: a randomized, double-blinded, placebo-controlled phase 3 trial. J Am Acad Dermatol 83(5):1282–1293. 10.1016/j.jaad.2020.06.054 32574587 10.1016/j.jaad.2020.06.054

[33] Paller AS, Simpson EL, Siegfried EC et al (2022) Dupilumab in children aged 6 months to younger than 6 years with uncontrolled atopic dermatitis: a randomised, double-blind, placebo-controlled, phase 3 trial. Lancet 400(10356):908–919. 10.1016/S0140-6736(22)01539-2 36116481 10.1016/S0140-6736(22)01539-2

[34] Tsai SYC, Gaffin JM, Hawryluk EB et al (2024) Evaluation of dupilumab on the disease burden in children and adolescents with atopic dermatitis: a population-based cohort study. Allergy 79(10):2748–2758. 10.1111/all.16265 39166365 10.1111/all.16265 PMC11608558

[35] Carnazza M, Werner R, Tiwari RK, Geliebter J, Li XM, Yang N (2025) The etiology of IgE-Mediated food allergy: potential therapeutics and challenges. Int J Mol Sci 26(4):1563. 10.3390/ijms26041563 40004029 10.3390/ijms26041563 PMC11855496

[36] Beck LA, Paller AS, Siegfried EC et al (2025) Dupilumab Treatment Significantly Reduces Age-Dependent Total IgE Levels in Young Children With Atopic Dermatitis. Presented at: 83rd Annual Meeting of the American Academy of Dermatology (AAD); March 7, ; Orlando, FL, USA

[37] van der Rijst LP, Hilbrands MS, Zuithoff NPA et al (2024) Dupilumab induces a significant decrease of food specific immunoglobulin E levels in pediatric atopic dermatitis patients. Clin Transl Allergy 14(7):e12381. 10.1002/clt2.12381 39019593 10.1002/clt2.12381 PMC11254451

[38] Emerson AG, Krantz MS, Robison RG (2025) Food allergy clinical course in children and adolescents treated with dupilumab for atopic dermatitis. Ann Allergy Asthma Immunol 135(5):555–559e1. 10.1016/j.anai.2025.07.023 40730273 10.1016/j.anai.2025.07.023 PMC12609014

[39] Starrenburg ME, Bel Imam M, Lopez JF et al (2024) Dupilumab treatment decreases MBC2s, correlating with reduced IgE levels in pediatric atopic dermatitis. J Allergy Clin Immunol 154(5):1333-1338.e4. 10.1016/j.jaci.2024.06.023 39038586 10.1016/j.jaci.2024.06.023

[40] Beck LA, Muraro A, Boguniewicz M, Chen Z, Zahn J, Rodríguez Marco A (2025) Dupilumab reduces inflammatory biomarkers in pediatric patients with moderate-to-severe atopic dermatitis. J Allergy Clin Immunol 155(1):135–143. 10.1016/j.jaci.2024.08.005 39178993 10.1016/j.jaci.2024.08.005

[41] Paller A, Guttman-Yassky E, Boguniewicz M, Levit N, Wolfe K (2022) 408 dupilumab reduces biomarkers indicative of type 2 inflammation in children aged ≥ 6 months to 5 years with moderate-to-severe atopic dermatitis. J Invest Dermatol 142(12):S250. 10.1016/j.jid.2022.09.421

[42] Zhang Y, Pi J, Wang L Dupilumab in Children Under 6 Years With Moderate-to-Severe Atopic Dermatitis: A 16-Week Real-World Prospective Study on Efficacy, Safety, and Local-Systemic Immune Responses. Allergy. n/a(n/a). 10.1111/all.70090 10.1111/all.70090 41055300

[43] van der Rijst L, Knol EF, van Wijk F et al (2024) 106 skin tape strip and serum biomarker profiles are reduced by dupilumab in pediatric atopic dermatitis. J Invest Dermatol 144(12):S246. 10.1016/j.jid.2024.10.112

[44] Wollenberg A, Blauvelt A, Guttman-Yassky E et al (2021) Tralokinumab for moderate‐to‐severe atopic dermatitis: results from two 52‐week, randomized, double‐blind, multicentre, placebo‐controlled phase III trials (ECZTRA 1 and ECZTRA 2). Br J Dermatol 184(3):437–449. 10.1111/bjd.19574 33000465 10.1111/bjd.19574 PMC7986411

[45] Guttman-Yassky E, Kabashima K, Staumont-Salle D et al (2024) Targeting IL-13 with tralokinumab normalizes type 2 inflammation in atopic dermatitis both early and at 2 years. Allergy 79(6):1560–1572. 10.1111/all.16108 38563683 10.1111/all.16108

[46] Silverberg JI, Guttman-Yassky E, Thaçi D et al (2023) Two phase 3 trials of lebrikizumab for moderate-to-severe atopic dermatitis. N Engl J Med 388(12):1080–1091. 10.1056/NEJMoa2206714 36920778 10.1056/NEJMoa2206714

[47] Guttman-Yassky E, Sun Z, Mena LR et al (2025) Lebrikizumab rapidly lowers inflammatory biomarkers with clinical correlations in moderate-to-severe atopic dermatitis. Dermatol Ther (Heidelb) 15(9):2595–2614. 10.1007/s13555-025-01481-4 40663228 10.1007/s13555-025-01481-4 PMC12354421

[48] Su JC, Spelman LJ, Eichenfield LF et al (2021) Efficacy and safety of crisaborole in patients aged 3 months to 17 years with mild-to-moderate atopic dermatitis. Pediatrics 147(3_MeetingAbstract):302–303. 10.1542/peds.147.3MA3.302

[49] Cooper KD, Kang K, Chan SC, Hanifin JM (1985) Phosphodiesterase inhibition by Ro 20-1724 reduces hyper-IgE synthesis by atopic dermatitis cells in vitro. J Invest Dermatol 84(6):477–482. 10.1111/1523-1747.ep12272486 3998494 10.1111/1523-1747.ep12272486

[50] Riaz F, Pan F, Wei P (2022) Aryl hydrocarbon receptor: the master regulator of immune responses in allergic diseases. Front Immunol 13:1057555. 10.3389/fimmu.2022.1057555 36601108 10.3389/fimmu.2022.1057555 PMC9806217

[51] FDA Approves Opzelura for Pediatric AD. Dermatology Education Foundation. September 19 (2025) Accessed December 11, 2025. https://dermnppa.org/fda-approves-opzelura-for-pediatric-ad/

[52] Kim BS, Howell MD, Sun K, Papp K, Nasir A, Kuligowski ME (2020) Treatment of atopic dermatitis with ruxolitinib cream (JAK1/JAK2 inhibitor) or triamcinolone cream. J Allergy Clin Immunol 145(2):572–582. 10.1016/j.jaci.2019.08.042 31629805 10.1016/j.jaci.2019.08.042

[53] Guttman-Yassky E, Teixeira HD, Simpson EL et al (2021) Once-daily upadacitinib versus placebo in adolescents and adults with moderate-to-severe atopic dermatitis (Measure Up 1 and Measure Up 2): results from two replicate double-blind, randomised controlled phase 3 trials. Lancet 397(10290):2151–2168. 10.1016/S0140-6736(21)00588-2 34023008 10.1016/S0140-6736(21)00588-2

[54] Hagino T, Hamada R, Yoshida M, Fujimoto E, Saeki H, Kanda N (2024) Total eosinophil count as a biomarker for therapeutic effects of upadacitinib in atopic dermatitis over 48 weeks. Front Immunol 15:1365544. 10.3389/fimmu.2024.1365544 38745653 10.3389/fimmu.2024.1365544 PMC11091278

[55] Torrelo A, Rewerska B, Galimberti M et al (2023) Efficacy and safety of baricitinib in combination with topical corticosteroids in paediatric patients with moderate-to-severe atopic dermatitis with an inadequate response to topical corticosteroids: results from a phase III, randomized, double-blind, placebo-controlled study (BREEZE-AD PEDS). Br J Dermatol 189(1):23–32. 10.1093/bjd/ljad096 36999560 10.1093/bjd/ljad096

[56] Guttman-Yassky E, Facheris P, Gomez-Arias PJ et al (2024) Effect of abrocitinib on skin biomarkers in patients with moderate-to-severe atopic dermatitis. Allergy 79(5):1258–1270. 10.1111/all.15969 38108208 10.1111/all.15969

[57] Ramsey N, Kazmi W, Phelan M, Lozano-Ojalvo D, Berin MC (2023) JAK1 inhibition with abrocitinib decreases allergen-specific basophil and T-cell activation in pediatric peanut allergy. J Allergy Clin Immunol Glob 2(3):100103. 10.1016/j.jacig.2023.100103 37711220 10.1016/j.jacig.2023.100103 PMC10501208

[58] *JAK Inhibition in Food Allergy*. clinicaltrials.gov; (2025) Accessed December 11, 2025. https://clinicaltrials.gov/study/NCT05069831

[59] Frazier W, Bhardwaj N (2020) Atopic dermatitis: diagnosis and treatment. Am Fam Physician 101(10):590–598 32412211

[60] Semmes EC, Chen JL, Goswami R, Burt TD, Permar SR, Fouda GG (2021) Understanding early-life adaptive immunity to guide interventions for pediatric health. Front Immunol. 10.3389/fimmu.2020.595297 33552052 10.3389/fimmu.2020.595297 PMC7858666

[61] Simon AK, Hollander GA, McMichael A (2015) Evolution of the immune system in humans from infancy to old age. Proc R Soc Lond B Biol Sci 282(1821):20143085. 10.1098/rspb.2014.3085 10.1098/rspb.2014.3085 PMC4707740 26702035

[62] Asrat S, Kaur N, Liu X et al (2020) Chronic allergen exposure drives accumulation of long-lived IgE plasma cells in the bone marrow, giving rise to serological memory. Sci Immunol 5(43):eaav8402. 10.1126/sciimmunol.aav8402 31924685 10.1126/sciimmunol.aav8402

[63] Limnander A, Kaur N, Asrat S et al (2023) A therapeutic strategy to target distinct sources of IgE and durably reverse allergy. Sci Transl Med 15(726):eadf9561. 10.1126/scitranslmed.adf9561 38091405 10.1126/scitranslmed.adf9561

[64] Murphy MB, Vitale L, O’Neill T et al (2025) Dual Inhibition of mast cells and thymic stromal lymphopoietin using a novel bispecific Antibody, CDX-622. Allergy 80(11):3115–3126. 10.1111/all.16509 39976188 10.1111/all.16509 PMC12590333

[65] Abdin R, Gharib R, Bunick CG, Issa NT (2024) Rapid remission of plaque psoriasis with bimekizumab treatment. J Drugs Dermatol 23(8):694–696. 10.36849/JDD.8381 39093648 10.36849/JDD.8381

[66] Mease PJ, Genovese MC, Weinblatt ME et al (2018) <article-title update="added"> Phase <scp>II</scp> Study of <scp>ABT</scp> ‐122, a Tumor Necrosis Factor– and Interleukin‐17A–Targeted Dual Variable Domain Immunoglobulin, in Patients With Psoriatic Arthritis With an Inadequate Response to Methotrexate. Arthritis Rheumatol 70(11):1778–1789. 10.1002/art.40579 29855175 10.1002/art.40579 PMC6221045

