# Correction to: Atopic Comorbidities and Topical Steroids in Early Childhood Atopic Dermatitis: Are We Missing a Piece of the Puzzle?

**DOI:** 10.1007/s12016-026-09144-8

**Published:** 2026-02-26

**Authors:** Courtney A. Chau, Sonya L. Cyr, Ruchi Gupta, Peter Lio

**Affiliations:** 1 Icahn School of Medicine at Mount Sinai, New York, NY USA; 2 Regeneron Pharmaceuticals Inc, Sleepy Hollow, NY USA; 3 Center for Food Allergy & Asthma Research, Institute for Public Health and Medicine, Northwestern University Feinberg School of Medicine, Chicago, IL USA; 4 Ann & Robert H. Lurie Children’s Hospital of Chicago, Chicago, IL USA; 5 Northwestern University Feinberg School of Medicine, 363 W. Erie Street, Suite #350, 60654 Chicago, IL USA

**Correction to: Clinical Reviews in Allergy & Immunology (2026) 69:3**.

10.1007/s12016-025-09131-5.

In this article, part of the caption for Figure 1 was mistakenly misplaced as the last paragraph in the ‘Conclusion’ section. The figure caption should have appeared as shown below.

**Fig. 1:**
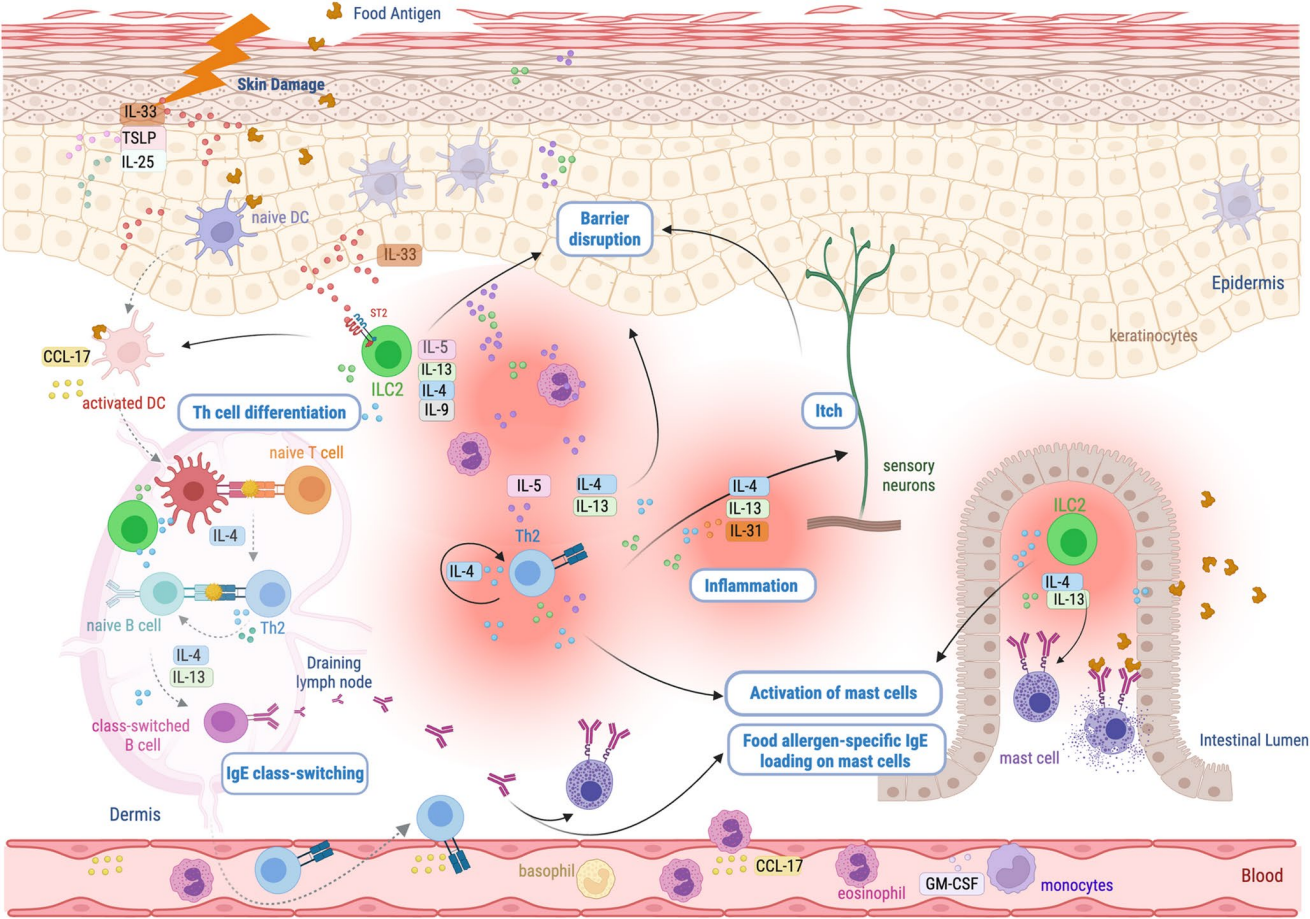
Graphical depiction of local and remote priming. Infant epicutaneous sensitization, or local priming, can occur through a combination epidermal damage, type 2 signaling and food antigen exposure. Epidermal damage is associated with alarmin signals (IL-33, IL-25 and TLSP) and ILC2 cytokines. Together, these promote DCs to educate memory T and B lymphocytes in a ‘danger’ context, thereby recognizing food antigens as allergens, resulting in the loading of skin-resident mast cells with allergen-specific antibodies. DCs and tissues also produce chemotactic agents such as CCL-17 enhancing the recruitment of type 2 effector cells from the circulation. Remote gut priming is thought to be initiated primarily with epidermal IL-33 binding of ST2 on ILC2s in a nonredundant manner for optimal distal IgE production. The role of ILC2s in this process may involve distinct mechanisms; 1-upstream of effector T cell generation by influencing DC migration or activation, 2-contribution to mast cell expansion in the intestine and 3-within lymphoid organs to complement the IL-4 and IL-13 primarily derived from T follicular helper (TFH) cells, thereby further enhancing B cell class switching to IgE. Gut resident mast cells are ultimately loaded with food-allergen specific IgE, primed for food allergen recognition and degranulation upon subsequent oral exposure. Created in Biorender [66].

The original article has been corrected.

